# Prevention of Postoperative Delirium in Patients Undergoing Elective Surgery Using Multicomponent Interventions: A Systematic Review and Meta‐Analysis of Randomized Controlled Trials

**DOI:** 10.1002/brb3.71131

**Published:** 2026-01-29

**Authors:** Xu Yang, Huachun Zhang, Sheng Peng

**Affiliations:** ^1^ Department of Anesthesiology Longhua Hospital Shanghai University of Traditional Chinese Medicine Shanghai China; ^2^ Department of Nursing Longhua Hospital Shanghai University of Traditional Chinese Medicine Shanghai China

**Keywords:** elective surgery, meta‐analysis, multicomponent interventions, postoperative delirium, systematic review

## Abstract

**Background and hypothesis:**

Postoperative delirium (POD) is a common acute cognitive disorder among older adults following elective surgery. It prolongs hospitalization, increases the risk of complications and readmission, and may contribute to long‐term cognitive decline, thereby reducing patients' quality of life. Although various preventive strategies have been developed, single interventions often yield limited efficacy. This study systematically evaluates, through meta‐analysis, the effectiveness of multicomponent interventions in preventing POD among older adults undergoing elective surgery.

**Study design:**

A systematic search was conducted in PubMed, Embase, Web of Science, and the Cochrane Library for randomized controlled trials (RCTs) published through July 2025. Risk ratios (RRs) with 95% confidence intervals (CIs) were calculated to assess intervention effects, and pooled analyses were performed using a random‐effects model.

**Study results:**

Eleven RCTs comprising 3857 patients undergoing elective surgery were included in the final analysis. Multicomponent interventions significantly reduced POD incidence in older adults (RR: 0.71; 95% CI: 0.59–0.85; *p* < 0.001), representing a 29% risk reduction. Between‐study heterogeneity was low (*I* ^2^ = 18.0%, *p* = 0.272). Sensitivity analyses supported the robustness of results. Subgroup analyses indicated a greater effect in Eastern populations compared to Western populations (interaction *p* = 0.045).

**Conclusions:**

Multicomponent interventions are effective in reducing POD incidence in older adults undergoing elective surgery, with geographical variation influencing effect size. These findings support broader clinical adoption of such interventions for POD prevention.

## Graphical abstract

We combined results from 11 high‐quality clinical trials involving 3857 older patients having planned surgery. Using a bundle of care methods significantly reduced the risk of developing delirium after surgery by 29%. The effect was stronger in studies from Eastern countries compared to Western ones.

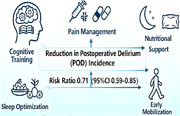

## 1 Background

With global population aging accelerating, the proportion of older adults undergoing elective surgery has increased markedly. At a global level, postoperative delirium (POD) is associated with a substantial economic burden, significantly increasing healthcare costs and resource utilization (Boone et al. 2020). Owing to the physiological stress induced by surgical trauma, combined with age‐related pathophysiological changes—including reduced physiological reserves, multimorbidity, and diminished cognitive capacity—this population is particularly susceptible to POD (Fernandez et al. 2025). POD is an acute neuropsychiatric syndrome characterized by sudden‐onset fluctuations in mental status, attention deficits, and global cognitive impairment (Mahanna‐Gabrielli et al. 2019; Inouye et al. 2014). POD incidence among older adults undergoing elective surgery varies widely (10%–50%) (Chen et al. 2024), influenced by factors such as the procedural invasiveness (Dilmen et al. 2024), anesthesia type (Wu et al. 2025), and patient's baseline conditions (de la Varga‐Martínez et al. 2025).

POD carries significant clinical consequences. In the short term, it is associated with 30%–50% longer hospital stays, a 2–3‐fold increase in postoperative complications, and higher 30‐day mortality rates (Gleason et al. 2015; Daiello et al. 2019; Harris et al. 2019). Long‐term studies identify POD as an independent risk factor for accelerated cognitive decline and a 40%–60% increased risk of dementia (Sturm et al. 2019; Ruck et al. 2024). These adverse outcomes impair patients' quality of life and drive up healthcare expenditures by an estimated 15%–20% annually, presenting major public health challenges (Vlisides and Avidan 2019).

Evidence‐based strategies for POD prevention include single‐modality interventions such as optimized analgesia, electrolyte and fluid balance, sleep enhancement, early mobilization, and cognitive stimulation (Du et al. 2025; Huang et al. 2025; Yang et al. 2025; Yuan et al. 2023; Kratz et al. 2015). However, given the complex pathophysiology of POD—which involves neuroinflammation, oxidative stress, cholinergic dysfunction, and circadian disruption—isolated interventions often prove insufficient (Paunikar and Chakole 2024; Nešković et al. 2025).

Multicomponent interventions offer an integrative approach aligned with the multifactorial etiology of POD. By targeting multiple biological and behavioral pathways simultaneously, they may yield superior preventive outcomes. In recent years, several randomized controlled trials (RCTs) have evaluated multicomponent strategies in older adults undergoing elective surgery; however, their findings vary due to heterogeneity in intervention protocols. This meta‐analysis aims to synthesize available RCTs to quantitatively assess the impact of multicomponent interventions on POD incidence in older adults undergoing elective surgery.

## 2 Materials and Methods

### 2.1 Data Sources, Search Strategy, and Selection Criteria

This systematic review and meta‐analysis was conducted in accordance with the 2020 Preferred Reporting Items for Systematic Reviews and Meta‐Analyses (PRISMA) guidelines (Page et al. 2021). Our study was registered in the INPLASY platform (number: INPLASY202570072). English‐language RCTs evaluating the effectiveness of multicomponent interventions for POD prevention in older adults were eligible for inclusion, regardless of publication status (published, in press, or ongoing). We searched PubMed, Embase, Web of Science, and the Cochrane Library through July 2025 using the following terms: (“postoperative delirium” [Mesh]) AND (“elective surgical procedures” [Mesh]) AND “randomized controlled trials” [Publication Type]. The full search strategies for all databases are provided in Appendix A. Additional studies were identified via ClinicalTrials.gov, and manual screening of reference lists from eligible studies was performed to capture potentially relevant publications.

Two investigators independently conducted study screening, data extraction, and quality assessment. Discrepancies were resolved through consensus with the first author. To minimize confounding and bias, inclusion was restricted to RCTs. Eligible studies met the following criteria: (1) population: patients aged 60 years or older undergoing any form of elective surgery. While the threshold of ≥65 years is common in clinical medicine, a lower threshold of ≥60 years was selected for this meta‐analysis to align with the definitions used in a significant portion of the included RCTs conducted in various international contexts and to maximize the inclusiveness of the available evidence; (2) intervention: multicomponent interventions; (3) comparator: standard care; (4) outcome: POD incidence; and (5) study design: RCT only. Studies were excluded if they (1) included patients undergoing emergency surgery; (2) evaluated a single‐component intervention only (e.g., pharmacology alone); (3) were published as abstracts, reviews, or case reports with insufficient data; or (4) did not report the primary outcome of postoperative delirium incidence.

### 2.2 Data Collection and Quality Assessment

Two reviewers independently extracted study data using a standardized form. Disagreements were resolved by group discussion. Extracted variables included the first author's surname, year of publication, country, sample size, proportion of male participants, mean age, surgical type, key components of multicomponent intervention, control group description, diagnostic tools for POD, and POD incidence. Risk of bias (RoB) was assessed using the Cochrane RoB tool (Higgins et al. 2011), which evaluates sequence generation, allocation concealment, blinding of participants and personnel, blinding of outcome assessment, incomplete outcome data, selective reporting, and other potential biases. A third reviewer resolved any disagreements after a full‐text review.

### 2.3 Statistical Analysis

Effect sizes were expressed as relative risks (RRs) with 95% confidence intervals (CIs) comparing multicomponent interventions to standard care on POD risk in older adults undergoing elective surgery. Due to anticipated heterogeneity in interventions and study populations, pooled estimates were calculated using a random‐effects model (DerSimonian and Laird 1986; Ades et al. 2005). Between‐study heterogeneity was assessed using the *I* ^2^ statistic and Cochran's *Q* test. An *I* ^2^ ≥ 50% indicated moderate‐to‐substantial heterogeneity, and a *p*‐value < 0.10 in Cochran's *Q* was considered significant (Deeks et al. 2008; Higgins et al. 2003). Sensitivity analysis was conducted by sequential exclusion of individual studies to evaluate result robustness (Tobias 1999). To investigate heterogeneity sources and intervention consistency, subgroup analyses were performed by geographic region, sample size, mean age, sex distribution, and surgical type. Interaction tests assessed between‐group differences (Altman and Bland 2003). Publication bias was examined using funnel plot symmetry and confirmed with Egger's and Begg's tests (Egger et al. 1997; Begg and Mazumdar 1994). All analyses were two‐sided, with statistical significance set at *p* < 0.05. Analyses were conducted using STATA 18.0 (StataCorp LP, USA).

## 3 Results

### 3.1 Literature Search

**FIGURE 1:**
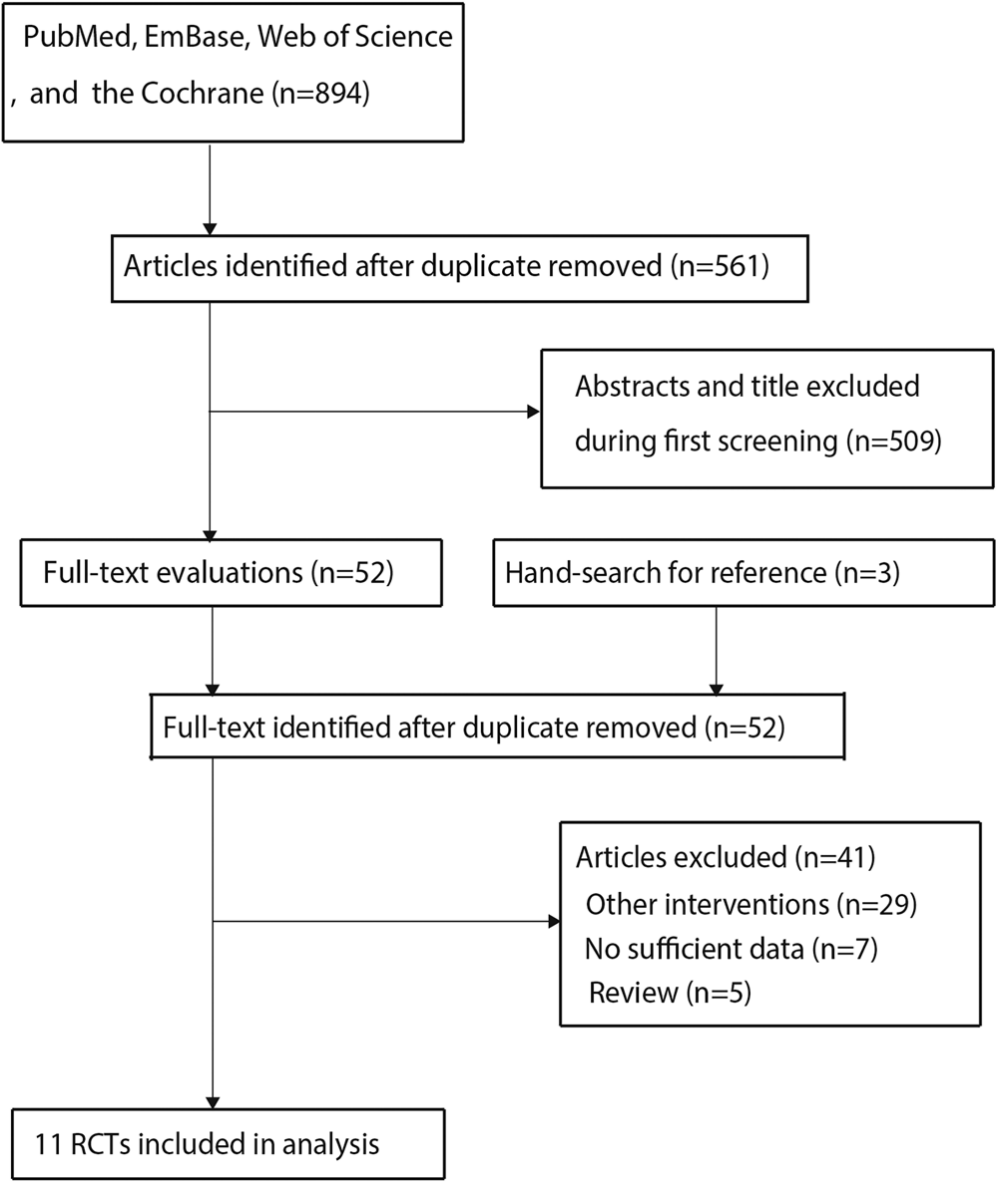
Flowchart of the literature search and study selection process.

The database search initially identified 894 records. After removing duplicates, 561 unique records remained. Title and abstract screening excluded 509 studies. A full‐text review of 52 articles led to the exclusion of 41: incompatible interventions (*n* = 29), lack of relevant outcomes (*n* = 7), and review articles (*n* = 5). Manual reference checks yielded three additional studies, all of which had already been identified. Ultimately, 11 RCTs met inclusion criteria and were included in the final meta‐analysis (Hempenius et al. 2013; Jia et al. 2014; Partridge et al. 2017; Chen et al. 2017; Vlisides et al. 2019; Humeidan et al. 2021; Deeken et al. 2022; Olotu et al. 2022; Lin et al. 2024; Jiang et al. 2024; Li et al. 2025). The study selection process is outlined in Figure 1.

### 3.2 Trials Characteristics

**FIGURE 2:**
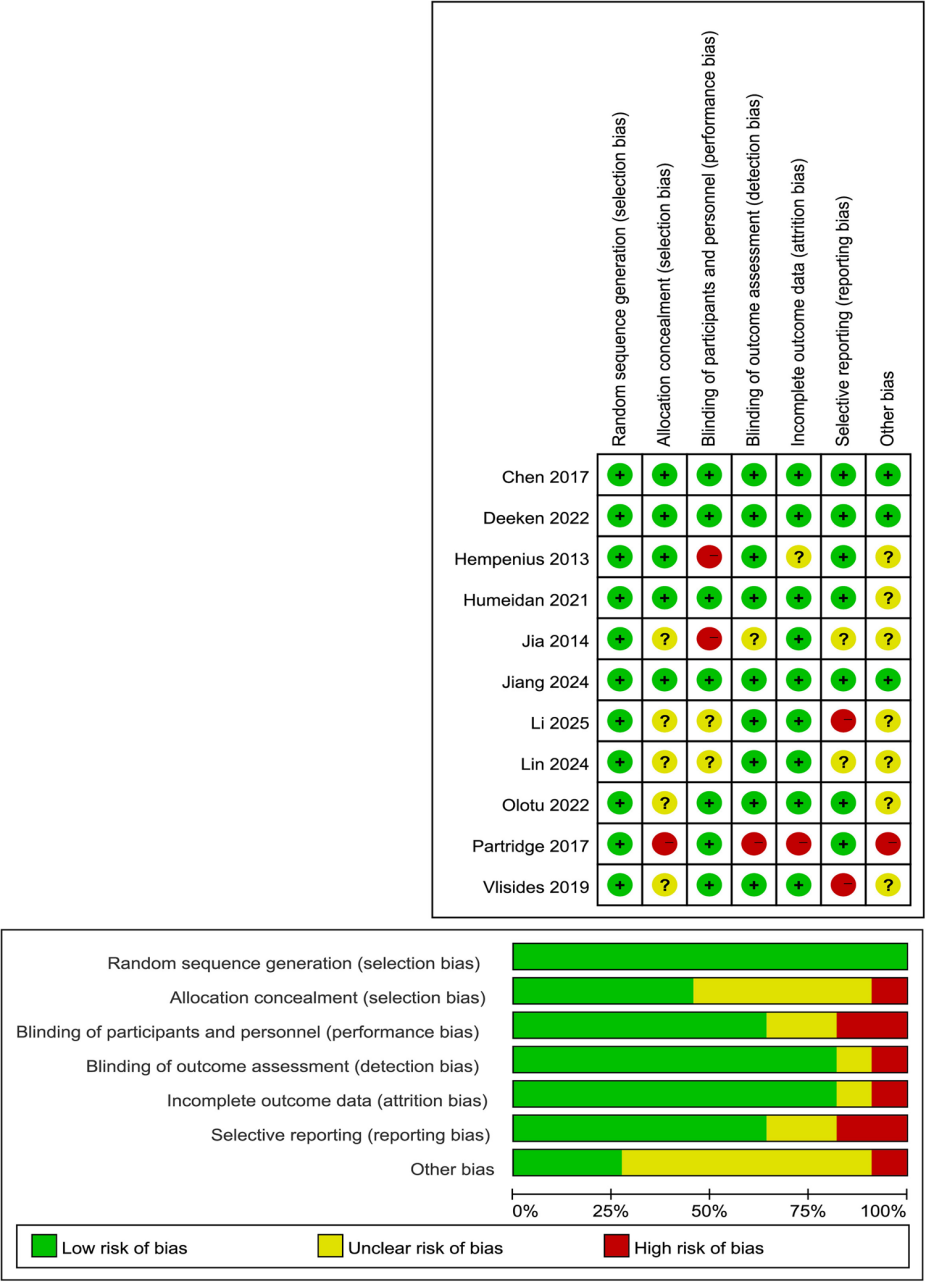
Risk of bias summary.

Table 1 presents the baseline characteristics of the 11 included RCTs, which enrolled a total of 3857 patients undergoing elective surgery. Sample sizes ranged from 52 to 1470. The mean age of participants ranged from 61.0 to 77.5 years, and the proportion of male participants ranged from 19.7% to 76.1%. As shown in Figure 2, most studies were rated as having a low risk of bias in the key methodological domains.

**TABLE 1:** The baseline characteristics of included studies and involved patients.

Study	Country	Sample size	Male (%)	Age (years)	Surgical procedure	Key components of multicomponent intervention	Control	Diagnostic tools for POD
Hempenius et al. (2013)	Netherlands	297	36.0	77.5	Cancer patients undergoing elective surgical procedure	Geriatric liaison intervention	Standard care	Delirium Observation Scale
Jia et al. (2014)	China	233	62.7	75.2	Open colorectal surgery	Fast‐track surgery	Standard care	Delirium Rating Scale‐Revised‐98
Partridge et al. (2017)	UK	209	76.1	75.5	Elective aortic aneurysm repair or lower‐limb arterial surgery	Preoperative comprehensive geriatric assessment and optimization	Standard care	Not assigned
Chen et al. (2017)	China	377	56.8	74.5	Abdominal surgery	Orienting communication, oral and nutritional assistance, and early mobilization	Standard care	Confusion Assessment Method
Vlisides et al. (2019)	USA	52	48.1	67.1	Scheduled non‐cardiac, non‐major vascular, or non‐intracranial surgery	Preoperative cognitive training regimen	Standard care	3DConfusion Assessment Method
Humeidan et al. (2021)	USA	251	35.1	67.0	Major non‐cardiac surgery	Electronic, tablet‐based preoperative cognitive exercise targeting memory, speed, attention, flexibility, and problem‐solving functions	Standard care	Confusion Assessment Method
Deeken et al. (2022)	Germany	1,470	51.9	78.0	Elective orthopedic, general, or cardiac surgery	Cognitive, motor, and sensory stimulation; meal companionship; accompaniment during diagnostic procedures; stress relaxation; and sleep promotion	Standard care	Confusion Assessment Method
Olotu et al. (2022)	Germany	558	34.1	71.8	Cardiovascular surgery	Delirium prevention bundle including reorientation measures, sleeping aids and early mobilization	Standard care	Confusion Assessment Method
Lin et al. (2024)	China	80	60.0	61.0	Cardiac valve surgery	Family intervention that instructed family caregivers to participate in delirium management during ICU visits.	Standard care	Confusion Assessment Method‐Intensive Care Unit
Jiang et al. (2024)	China	208	69.2	65.5	Cardiac surgery	Cognitive training, which included substantial practice with online tasks designed to enhance cognitive functions including memory, imagination, reasoning, reaction time, attention, and processing speed	Standard care	Confusion Assessment Method or Confusion Assessment Method‐Intensive Care Unit
Li et al. (2025)	China	122	19.7	71.0	Total hip and knee arthroplasty	Enhance visuospatial abilities, reaction speed, and memory	Standard care	3DConfusion Assessment Method

**Abbreviation**: POD, postoperative delirium.

### 3.3 Meta‐Analysis

**FIGURE 3:**
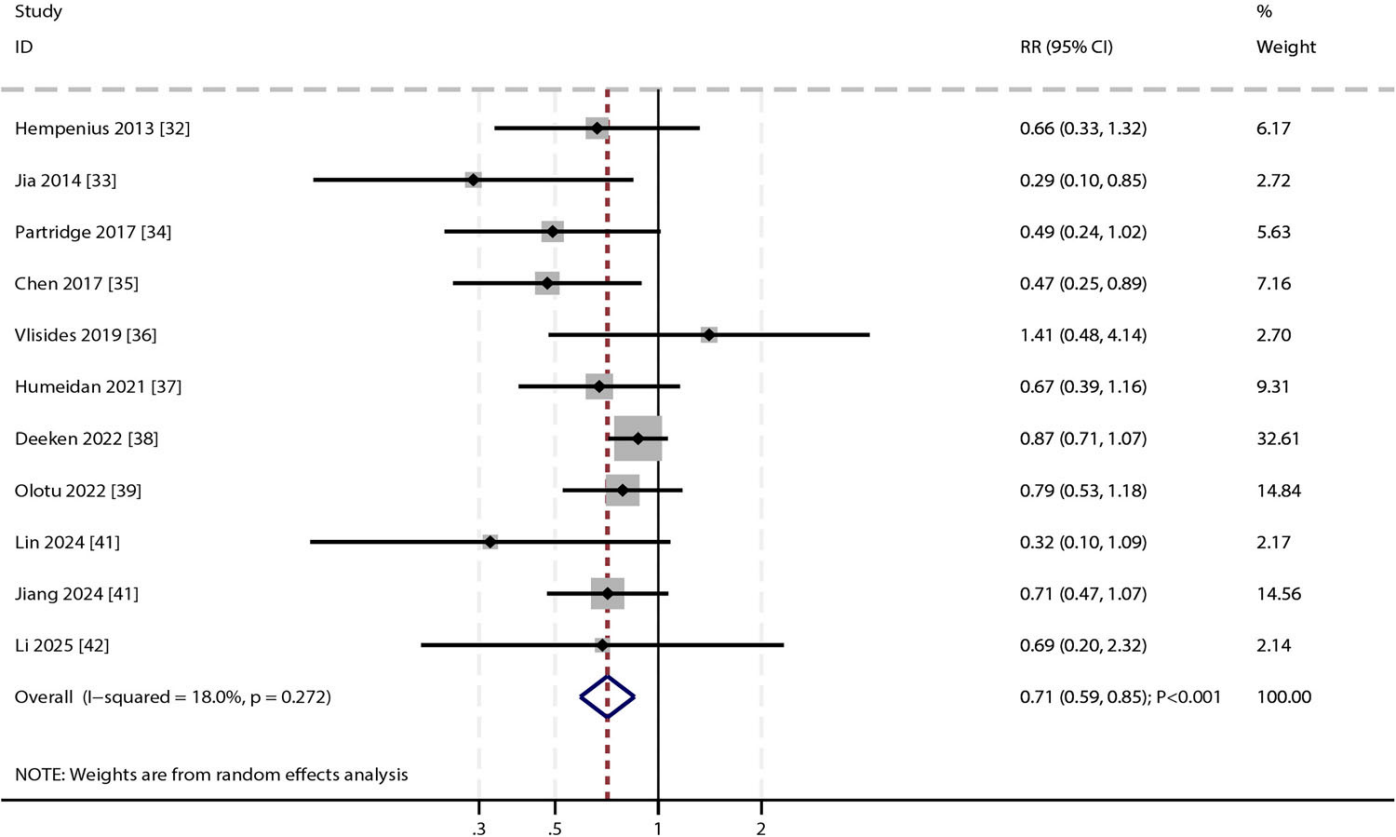
Effect of multicomponent interventions vs. standard care on the risk of postoperative delirium.

Pooled analysis showed that multicomponent interventions significantly reduced POD incidence among older adults undergoing elective surgery compared with standard care (RR: 0.71; 95% CI: 0.59–0.85; *p* < 0.001; Figure 3), corresponding to a 29% relative risk reduction. Heterogeneity was low (*I* ^2^ = 18.0%, *p* = 0.272), suggesting minimal influence from variability in intervention designs or study populations.

### 3.4 Sensitivity Analysis

**FIGURE 4:**
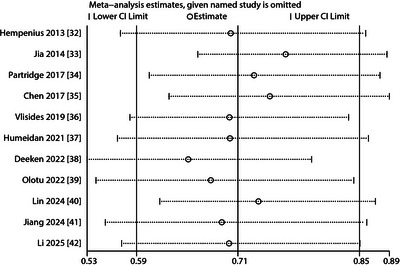
Sensitivity analysis of the effect of multicomponent interventions on postoperative delirium risk.

To assess the robustness of our findings, we conducted sensitivity analyses by sequentially excluding each study and recalculating the pooled RR with 95% CIs for multicomponent interventions versus standard care. The results indicated consistent risk reduction, as all recalculated RRs remained <1 with 95% CIs excluding 1 (Figure 4). This confirms that the primary conclusion—that multicomponent interventions reduce POD risk in older adults undergoing surgery—is not substantially influenced by any single study, underscoring the reliability of our findings.

### 3.5 Subgroup Analysis

Subgroup analyses showed that multicomponent interventions significantly reduced POD risk in most predefined subgroups compared with standard care. However, this protective effect did not reach statistical significance in studies with sample sizes ≥300. Notably, the magnitude of POD risk reduction was significantly greater in Eastern populations than in Western populations (ratio of RR: 1.42; 95% CI: 1.00–2.01; *P*_interaction_ = 0.045) (Table 2).

**TABLE 2:** Subgroup analyses for POD.

Factors	Subgroups	RR and 95%CI	*p*‐value	*I* ^2^ (%)	*Q* statistic	Interaction *P* test	RRR
Country	Western	0.81 (0.69–0.95)	0.011	0.0	0.526	0.045	1.42 (1.00–2.01)
	Eastern	0.57 (0.42–0.78)	< 0.001	0.6	0.403		
Sample size	≥ 300	0.77 (0.59‐1.02)	0.064	39.3	0.193	0.104	1.20 (0.83‐1.74)
	< 300	0.64 (0.50‐0.82)	< 0.001	0.0	0.510		
Mean age (years)	≥ 70	0.69 (0.54‐0.88)	0.003	31.2	0.190	0.603	0.99 (0.66‐1.48)
	< 70	0.70 (0.51‐0.97)	0.032	6.6	0.360		
Male (%)	≥ 60	0.53 (0.36‐0.80)	0.003	20.5	0.287	0.071	0.66 (0.43‐1.02)
	< 60	0.80 (0.69‐0.94)	0.006	0.0	0.524		
Surgical type	Cardiac surgery	0.68 (0.53–0.89)	0.004	0.0	0.426	0.370	0.96 (0.65–1.40)
	Major non‐cardiac surgery	0.71 (0.53–0.93)	0.014	30.3	0.197		

### 3.6 Publication Bias

**FIGURE 5:**
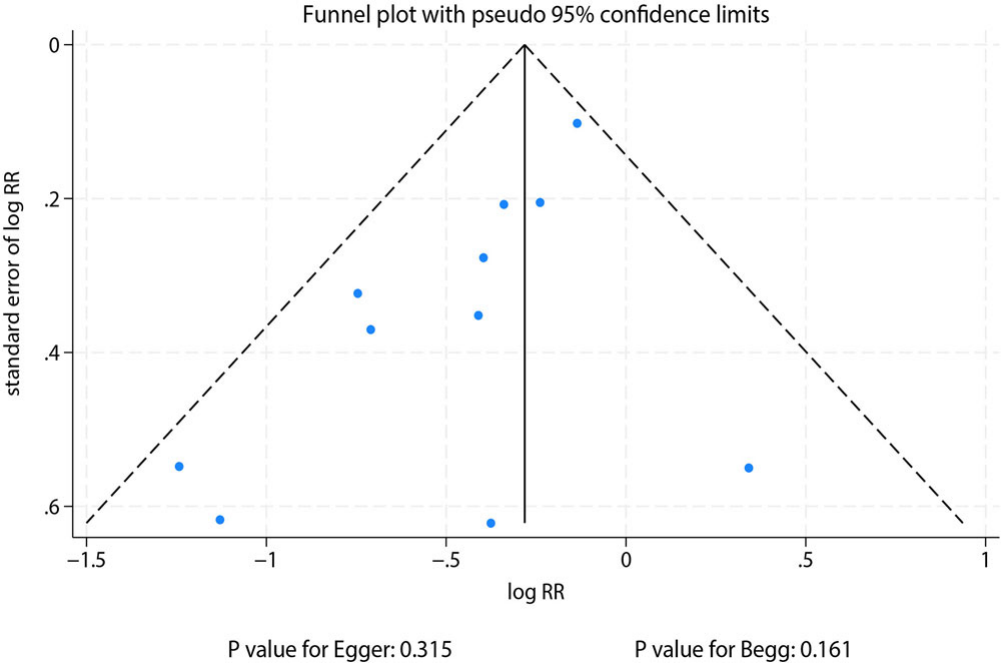
Funnel plot assessing publication bias for studies reporting postoperative delirium risk.

Although visual inspection of the funnel plot could not entirely exclude potential publication bias, quantitative assessments indicated no significant bias (Egger's test *p* = 0.315; Begg's test *p* = 0.161; Figure 5).

## 4 Discussion

This systematic review and meta‐analysis synthesized data from 11 RCTs involving 3,857 patients and demonstrated that multicomponent interventions significantly reduce POD incidence in older adult surgical patients, corresponding to a 29% relative risk reduction. These findings exhibited high robustness and consistency. The results not only provide Level I evidence for POD prevention in clinical settings but also contribute to understanding the multifactorial pathogenesis of POD and inform perioperative management strategies.

Our findings align with recent clinical studies on multicomponent approaches. For example, in hip fracture surgery, integrated protocols incorporating pain management, sleep optimization, and early mobilization reduced POD risk by over 30% (Janssen et al. 2019), mirroring our pooled effect size. This consistency suggests that, regardless of surgical type, multicomponent interventions exert synergistic effects by targeting multiple POD pathways simultaneously, including neuroinflammation, cholinergic dysfunction, and circadian rhythm disruption. In contrast to single‐modal interventions (e.g., analgesia or fluid optimization alone), multicomponent strategies address POD's complex pathophysiology more comprehensively (Nešković et al. 2025), explaining the limited efficacy of unimodal approaches due to their narrow scope (Paunikar and Chakole 2024). Notably, despite variation in specific intervention elements across the 11 studies, the low heterogeneity supports stable preventive effects. This reinforces the core principle of multicomponent interventions: meaningful risk reduction can be achieved by addressing key POD risk factors, even without strict standardization. Such flexibility is essential for real‐world implementation, allowing care teams to tailor interventions to individual patient characteristics, thereby enhancing clinical feasibility.

Subgroup analyses revealed significantly greater POD risk reduction in Eastern versus Western populations. Several factors may contribute to this disparity: (1) Differences in healthcare delivery—Eastern systems’ emphasis on holistic care may promote better adherence to multicomponent protocols (Song et al. 2025); (2) Genetic predisposition—lower prevalence of the APOE ε4 allele, a known POD risk factor, in Asian populations may enhance intervention effectiveness (Xu et al. 2025; Vasunilashorn et al. 2020); and (3) Cultural influences on acceptance of intervention components such as early mobilization and sleep management. Additionally, studies with sample sizes ≥300 did not show significant effects. This is a recognized phenomenon in implementation science, where the scale‐up from smaller, tightly controlled efficacy trials to larger, more pragmatic effectiveness trials can lead to a dilution of the intervention effect. In larger, multi‐center studies, maintaining uniform, high‐fidelity implementation of complex, multicomponent interventions across all clinical sites and staff is challenging. Protocol adherence may be more variable, and the intensity of individual intervention components may be inadvertently weakened compared to smaller, single‐center trials where closer supervision is feasible. This ‘performance bias’ at scale could contribute to the observed attenuation of the effect. Furthermore, larger trials often employ broader inclusion criteria to enhance generalizability, which increases baseline patient heterogeneity and may also obscure a true treatment effect present in more specific subpopulations.

These findings offer practical, evidence‐based recommendations for POD prevention in older adults undergoing surgery. Multicomponent interventions should replace single‐modal strategies. Based on consistent evidence, the core intervention bundle should include (1) optimized analgesia using opioid‐sparing techniques (Du et al. 2025); (2) fluid and nutritional support (Chen et al. 2017); (3) structured sleep protocols (e.g., minimizing nighttime disruptions, melatonin administration) (Yuan et al. 2023); (4) early graded mobilization (Olotu et al. 2022); and (5) daily cognitive stimulation (e.g., orientation exercises, interactive games) (Kratz et al. 2015). Data from Eastern populations suggest that incorporating regional practices such as acupoint stimulation and family‐integrated care may further improve outcomes. Given that POD‐related costs account for 15%–20% of annual healthcare expenditures (Vlisides and Avidan 2019), the 29% risk reduction demonstrated in this analysis underscores the potential for both clinical benefit and substantial cost savings.

Several limitations should be acknowledged. First, although between‐study heterogeneity was low, variability in intervention components may affect the precision of effect size estimates. Second, while Egger and Begg's tests did not detect significant publication bias, minor funnel plot asymmetry suggests possible underreporting of negative results. Third, no included studies differentiated POD subtypes, limiting our ability to evaluate subtype‐specific efficacy. Finally, the absence of long‐term follow‐up data restricts assessment of the interventions’ impact on cognitive outcomes associated with POD. Furthermore, future trials should prioritize the collection of long‐term cognitive outcomes to determine if reducing POD incidence translates into sustained cognitive benefits. A standardized 3‐month follow‐up assessment using the Telephone Montreal Cognitive Assessment is a feasible and validated method to track cognitive trajectories and should be incorporated as a key secondary outcome in future studies.

## 5 Conclusion

In conclusion, this meta‐analysis provides robust evidence supporting the integration of multicomponent interventions into standard perioperative care for older adults to mitigate POD risk. To translate this evidence into widespread practice and further refine preventive strategies, future research should focus on three key priorities. First, there is a need to optimize intervention bundles for cost‐effectiveness; while our results show a significant clinical benefit, formal economic analyses are required to demonstrate the value proposition to healthcare systems and facilitate funding and adoption. Second, future work must focus on standardizing implementation pathways to ensure fidelity and efficacy across diverse clinical settings, addressing the logistical challenges that often hinder the consistent application of complex, multicomponent protocols. Finally, and most prospectively, we highlight the potential of exploring artificial intelligence (AI) and machine learning approaches for personalized POD prevention. The multifactorial etiology of POD makes it an ideal use case for AI. By integrating preoperative electronic health record data, genetic markers, and real‐time physiological parameters, AI models could stratify patients into precise risk categories, enabling the targeted deployment of intensive multicomponent interventions to those who would benefit most, thereby optimizing resource allocation and improving overall preventive efficacy.

## Author Contributions

**Xu Yang**: conceptualization, data curation, investigation, visualization, writing – original draft, writing – review and editing. **Huachun Zhang, Sheng Peng**: data curation, investigation, writing – original draft, writing – review and editing.

## Funding

The authors have nothing to report.

## Conflicts of Interest

The authors declare no conflicts of interest.

## Supporting information




**Supplementary Materials**: brb371131‐sup‐0001‐AppendixA.docx

## Data Availability

All data, models, and code generated or used during the study appear in the submitted article.
