# Supplementary material for: Prevention of Postoperative Delirium in Patients Undergoing Elective Surgery Using Multicomponent Interventions: A Systematic Review and Meta‐Analysis of Randomized Controlled Trials
Source: Brain Behav. 2026 Jan 29;16(2):e71131. doi: 10.1002/brb3.71131 (PMC12856225; doi:10.1002/brb3.71131)
Supplement: Supplementary file 1 — Supplementary Materials: brb371131‐sup‐0001‐AppendixA.docx [file BRB3-16-e71131-s001.docx]

**Complete Search Strategies for All Databases**

**PubMed:**

("postoperative delirium"[Mesh] OR "postoperative delirium"[tiab] OR "post-operative delirium"[tiab] OR "post-surgical delirium"[tiab] OR "delirium, postoperative"[tiab] OR POD[tiab])

AND

("elective surgical procedures"[Mesh] OR "elective surgery"[tiab] OR "elective surgical"[tiab] OR "elective procedure*"[tiab] OR "scheduled surgery"[tiab] OR non-emergency surgery[tiab])

AND

("randomized controlled trial"[pt] OR "controlled clinical trial"[pt] OR randomized[tiab] OR randomised[tiab] OR placebo[tiab] OR "clinical trials as topic"[mesh:nocxp] OR randomly[tiab] OR trial[ti])

**Embase:**

1. 'postoperative delirium'/exp OR 'postoperative delirium':ti,ab OR 'post-operative delirium':ti,ab OR 'post-surgical delirium':ti,ab OR 'delirium, postoperative':ti,ab OR 'pod':ti,ab

2. 'elective surgery'/exp OR 'elective surgery':ti,ab OR 'elective surgical':ti,ab OR 'elective procedure*':ti,ab OR 'scheduled surgery':ti,ab OR 'non-emergency surgery':ti,ab

3. 'randomized controlled trial'/exp OR 'randomized controlled trial':ti,ab OR 'randomised controlled trial':ti,ab OR 'randomized':ti,ab OR 'randomised':ti,ab OR 'placebo':ti,ab OR 'clinical trial':ti,ab OR 'randomly':ti,ab OR 'trial':ti

4. 1 AND 2 AND 3

**Web of Science:**

TS=("postoperative delirium" OR "post-operative delirium" OR "post-surgical delirium" OR "delirium, postoperative" OR POD)

AND

TS=("elective surgery" OR "elective surgical" OR "elective procedure*" OR "scheduled surgery" OR "non-emergency surgery")

AND

TS=(randomized OR randomised OR "randomized controlled trial" OR "randomised controlled trial" OR placebo OR "clinical trial" OR randomly OR trial)

**Cochrane Library:**

#1 MeSH descriptor: [Postoperative Delirium] explode all trees

#2 (postoperative delirium OR post-operative delirium OR post-surgical delirium OR delirium, postoperative OR POD):ti,ab,kw

#3 #1 OR #2

#4 MeSH descriptor: [Elective Surgical Procedures] explode all trees

#5 (elective surgery OR elective surgical OR elective procedure* OR scheduled surgery OR non-emergency surgery):ti,ab,kw

#6 #4 OR #5

#7 #3 AND #6

#8 Publication Type: Randomized Controlled Trial

#9 #7 AND #8
